# A meta-analysis to derive literature-based benchmarks for readmission and hospital mortality after patient discharge from intensive care

**DOI:** 10.1186/s13054-014-0715-6

**Published:** 2014-12-31

**Authors:** F Shaun Hosein, Derek J Roberts, Tanvir Chowdhury Turin, David Zygun, William A Ghali, Henry T Stelfox

**Affiliations:** Department of Community Health Sciences, University of Calgary, 3280 Hospital Drive NW, Calgary, Canada; Department of Surgery, University of Calgary, 3280 Hospital Drive NW, Calgary, AB T2N 4Z6 Canada; Department of Critical Care Medicine, University of Calgary, 3280 Hospital Drive NW, Calgary, AB T2N 4Z6 Canada; Department of Critical Care Medicine, Alberta Health Services, 11220-83 Ave, Edmonton, AB T6G 2B7 Canada; Division of Critical Care, University of Alberta, 11220-83 Ave, Edmonton, AB T6G 2B7 Canada; Department of Medicine, University of Calgary, 3280 Hospital Drive NW, Calgary, AB Canada

## Abstract

**Introduction:**

We sought to derive literature-based summary estimates of readmission to the ICU and hospital mortality among patients discharged alive from the ICU.

**Methods:**

We searched MEDLINE, Embase, CINAHL and the Cochrane Central Register of Controlled Trials from inception to March 2013, as well as the reference lists in the publications of the included studies. We selected cohort studies of ICU discharge prognostic factors that in which readmission to the ICU or hospital mortality among patients discharged alive from the ICU was reported. Two reviewers independently abstracted the number of patients readmitted to the ICU and hospital deaths among patients discharged alive from the ICU. Fixed effects and random effects models were used to estimate the pooled cumulative incidence of ICU readmission and the pooled cumulative incidence of hospital mortality.

**Results:**

The analysis included 58 studies (*n* = 2,073,170 patients). The majority of studies followed patients until hospital discharge (*n* = 46 studies) and reported readmission to the ICU (*n* = 46 studies) or hospital mortality (*n* = 49 studies). The cumulative incidence of ICU readmission was 4.0 readmissions (95% confidence interval (CI), 3.9 to 4.0) per 100 patient discharges using fixed effects pooling and 6.3 readmissions (95% CI, 5.6 to 6.9) per 100 patient discharges using random effects pooling. The cumulative incidence of hospital mortality was 3.3 deaths (95% CI, 3.3 to 3.3) per 100 patient discharges using fixed effects pooling and 6.8 deaths (95% CI, 6.1 to 7.6) per 100 patient discharges using random effects pooling. There was significant heterogeneity for the pooled estimates, which was partially explained by patient, institution and study methodological characteristics.

**Conclusions:**

Using current literature estimates, for every 100 patients discharged alive from the ICU, between 4 and 6 patients on average will be readmitted to the ICU and between 3 and 7 patients on average will die prior to hospital discharge. These estimates can inform the selection of benchmarks for quality metrics of transitions of patient care between the ICU and the hospital ward.

## Introduction

Transitions of patient care between providers are vulnerable periods in health care delivery that expose patients to preventable errors and adverse events [1]. The discharge of patients from the intensive care unit (ICU) to a hospital ward is one of the highest-risk transitions of care [1]. This has been attributed to the sickest patients in the hospital being transitioned from a resource-rich environment to one with fewer resources, the number of providers involved, a lack of standardized discharge procedures and the complexity of verbal and written communication between providers and patients and/or their families as well as between providers themselves [2-5].

Opportunities exist to improve the quality of care during ICU discharge, and measures of ICU readmission and hospital mortality following patient discharge from the ICU have been proposed as quality metrics [6-10]. However, the reported incidences of readmission and hospital mortality vary widely, and there are currently no established benchmarks to guide quality improvement efforts [11,12].

Therefore, we performed a secondary meta-analysis of studies by conducting a systematic review of prognostic factors for readmission to the ICU and hospital mortality in patients discharged alive from the ICU to derive literature-based estimates of these outcomes.

## Material and methods

We followed the recommendations set forth in the Preferred Reporting Items in Systematic Reviews and Meta-Analysis and Meta-Analysis of Observational Studies in Epidemiology statements [13,14]. This study did not require research ethics approval, as all of the data are in the public domain. Similarly, no consent was required from patients, as all of the data were abstracted in aggregate and are available in the public domain.

### Search strategy and data sources

We systematically searched the following four databases for articles published between the inception dates of the databases and March 2013: MEDLINE, Embase, CINAHL and Cochrane Central Register of Controlled Trials. Searches were completed using a combination of the following terms: “intensive care unit,” “patient discharge” and readmission/mortality/medical emergency team activation, with appropriate wildcards and variations in spelling. We identified additional articles by reviewing the reference lists of studies identified for inclusion.

### Inclusion criteria

We selected all studies in which prognostic factors for ICU readmission and hospital mortality were reported. The following were the inclusion criteria: (1) study design was a cohort study, (2) study participants were adult patients (>16 years old) who were discharged alive from the ICU, (3) prognostic factors for ICU discharge were reported and (4) raw data were reported that allowed calculation of the cumulative incidence of ICU readmission or the cumulative incidence of hospital mortality for patients discharged alive from the ICU prior to hospital discharge. Because there is no widely accepted time period for measuring readmission and mortality after patient discharge from the ICU (for example, 24 hours), and because authors of previous reviews have reported the use of different time periods, we included all follow-up periods [15]. We excluded articles that described discharge from a high-dependency or step-down unit. Two reviewers independently and in duplicate reviewed the titles and abstracts of retrieved publications and subsequently the full text of relevant articles. Agreement between reviewers for inclusion of full-text articles was good (κ = 0.84, 95% confidence interval (CI), 0.67 to 1.00).

### Data abstraction

Two reviewers independently and in duplicate abstracted data describing study purpose, design, setting (country, type of ICU), sample size, study population (age, length of follow-up, severity of illness), outcomes (readmission to the ICU and hospital mortality following patient discharge alive from the ICU) and study quality. Disagreements were resolved through consensus. Authors of the included studies were contacted to gather missing data.

### Risk of bias assessment

Study quality was evaluated using 11 characteristics: ethical approval reported, eligibility criteria described, definition of cohort timing provided, demographics described, comorbidities reported, severity of illness score reported, study duration reported, completeness of follow-up, adjustment for potential confounders, sample size calculation reported and study limitations reported. Studies that satisfied six or more of the criteria were classified as being of high quality.

### Analysis

In the primary analysis, we focused on describing the cumulative incidence of readmission to the ICU and the cumulative incidence of hospital mortality for patients discharged alive from the ICU. Readmissions to the ICU and hospital mortality were calculated using data from each article on raw events (total number of events) and study population (total number of patients discharged alive from the ICU). The cumulative incidence was pooled using both Mantel-Haenszel fixed effects (assumes a single common incidence across studies) and DerSimonian and Laird random effects models (does not assume a single common incidence across studies) [16,17].

Statistical heterogeneity was examined by calculating *I*^2^-statistics, wherein a *P*-value <0.05 and an *I*^2^-value >50% indicated the presence of heterogeneity among the included studies [18]. Stratified analyses were performed to examine for potential sources of heterogeneity between studies using prespecified subgroups that included geographic region (North America, Europe, Australasia, other region), ICU type (medical-surgical, cardiovascular, other ICU), patient characteristics (age <60 years vs. ≥60 years, predicted mortality <10% vs. ≥10% according to illness severity score) and study characteristics (patients with do-not-resuscitate (DNR) goals of care included, adjustment for confounding factors, duration of follow up ≤21 days vs. >21 days, sample size <1,000 patients vs. ≥1,000 patients, number of ICUs 1 vs. >1 and a composite measure of study quality).

All data analysis was conducted using Stata version 11.0 software (StataCorp, College Station, TX, USA).

## Results

**Figure 1:**
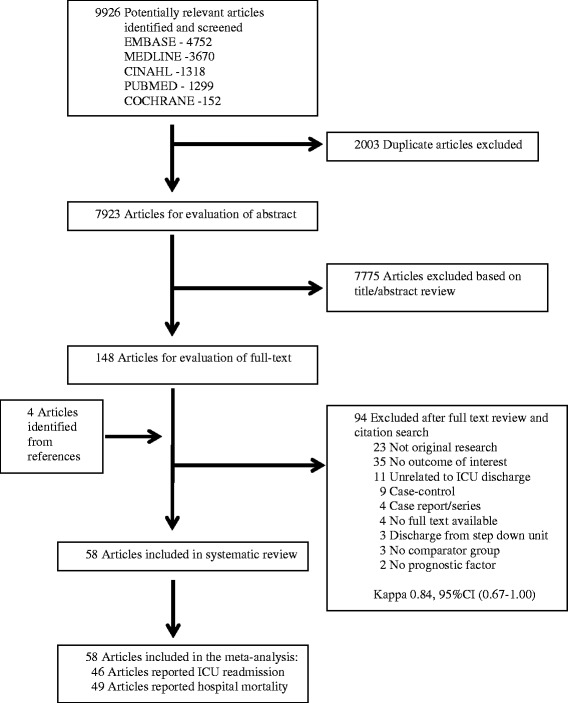
**Selection process for articles for review.** CI, Confidence interval; ICU, Intensive care unit.

We identified 58 studies that satisfied the inclusion criteria and that had data which allowed calculation of the cumulative incidence of readmission to the ICU (*n* = 46 studies) or the cumulative incidence of hospital mortality (*n* = 49 studies) for patients discharged alive from the ICU (Figure 1) [2,4,5,8,11,12,19-70]. The characteristics of the studies are summarized in Table 1. The studies were published between 1986 and 2013 and represented 18 countries, including the United States (*n* = 12), the United Kingdom (*n* = 8), Australia (*n* = 6), Canada (*n* = 6) and Germany (*n* = 4). The number of patients within the studies ranged from 86 to 704,963, with an aggregate total of 2,073,170 patients included in our meta-analysis. The majority of studies were conducted in mixed medical-surgical ICUs (*n* = 34), with fewer studies conducted in cardiac ICUs (*n* = 7) or exclusively medical ICUs (*n* = 4) or surgical ICUs (*n* = 3). The mean (standard deviation) age of patients was 59.7 (5.4) years among the 44 studies in which a mean age was reported. Patient illness severity in most studies was reported based on the Acute Physiology and Chronic Health Evaluation score (*n* = 31) or the Simplified Acute Physiology Score (*n* = 12). The majority of studies were single-centered (*n* = 32), included patients with DNR orders (*n* = 42) and used multivariable adjustment (*n* = 49) in their data analysis. Most studies followed patients until hospital discharge (*n* = 46). In three studies, the investigators reported readmission to the ICU and hospital mortality at fixed time periods following patient discharge from the ICU (48 hours [26], 7 days [44] and 2 weeks [36]).

**Table 1:** **Description of included studies** ^**a**^

**Study**	**Year**	**Countries**	**Follow-up**	**Type of ICU**	**ICUs,** ***n***	**Patients,** ***n***	**Age, yr (mean)**	**Female (%)**	**SOI measure**	**SOI score (mean)**	**Readmission (%)**	**Mortality (%)**
Strauss *et al.* [70]	1986	USA	Hospital discharge	Medical-surgical	1	912	50	N/A	APS	N/A	15	9.9
Rubins *et al.* [69]	1988	USA	Hospital discharge	Medical	1	229	59.9	2.2	APACHE II	10.6	13.1	3
Chen *et al.* [68]	1998	Canada	Hospital discharge	Medical-surgical	7	5,127	59.3	38.0	APACHE II	17.1	4.6	5.5
Cohn *et al.* [67]	1999	USA	Hospital discharge	Cardiovascular	38	2,228	65.3	32.4	N/A	N/A	5.7	1.0
Cooper *et al.* [8]	1999	USA	Hospital discharge	Various^c^	28	103,968	63.5	48.0	APACHE III	44.3	6.1	N/A
Smith *et al.* [66]	1999	UK	N/A	Medical-surgical	1	283	66	45.6	APACHE II	17^b^	7.8	11
Goldfrad and Rowan [65]	2000	UK	Hospital discharge	Medical-surgical	62	12,748	58.2	N/A	APACHE II	14.7	8.3	17.1
Daly *et al.* [64]	2001	UK	Hospital discharge	Medical-surgical	1	5,475	N/A	30.5	APACHE II	13.7	2.6	3.7
Rosenberg *et al.* [5]	2001	USA	Hospital discharge	Medical	1	3,310	53	66.5	APACHE III	49	9.6	9.6
Moreno *et al.* [63]	2001	Netherlands	Hospital discharge	N/A	48	2,958	N/A	N/A	SAPS II	30.1	N/A	8.6
Calafiore *et al.* [61]	2002	Italy	Hospital discharge	Cardiovascular	1	1,194	N/A	18.5	N/A	N/A	1.3	0.3
Beck *et al.* [62]	2002	UK	Hospital discharge	Medical-surgical	1	1,654	57	38.3	APACHE II	18.3	7.6	12.6
Kogan *et al.* [58]	2003	Israel	Hospital discharge	N/A	1	1,613	63.5	N/A	N/A	N/A	3.3	0.4
Bardell *et al.* [59]	2003	Canada	Hospital discharge	Cardiovascular	1	2,117	65	30.0	N/A	N/A	3.5	2.8
Metnitz *et al.* [57]	2003	Austria	Hospital discharge	Medical-surgical	30	15,180	62.7	39.4	N/A	N/A	5.1	N/A
Uusaro *et al.* [56]	2003	Finland	Hospital discharge	N/A	18	20,636	N/A	N/A	SAPS II	34	N/A	10.1
Azoulay *et al.* [60]	2003	France	Hospital discharge	Various^d^	7	1,385	65^b^	36.5	SAPS II	36^b^	N/A	10.8
Yoon *et al.* [53]	2004	Korea	Hospital discharge	Medical-surgical	34	1,929	55.5	35.8	APACHE III	N/A	4.1	17.3
Duke *et al.* [55]	2004	Australia	Hospital discharge	Medical-surgical	1	1,870	62^b^	N/A	APACHE II	18.5	5.1	4.9
Fortis *et al.* [54]	2004	Greece	Hospital discharge	Medical-surgical	1	86	63	43.0	APACHE II	14	N/A	15.1
Vohra *et al.* [52]	2005	UK	Hospital discharge	Cardiovascular	1	7,177	70.4	N/A	N/A	N/A	2.5	N/A
Azoulay *et al.* [2]	2005	Europe, Canada, Israel	Hospital discharge	Medical-surgical	28	1,872	60^b^	37.4	SAPS II	35^b^	N/A	10.4
Alban *et al.* [51]	2006	USA	Hospital discharge	Surgical	1	10,840	58.8	N/A	APACHE II	15.4	2.7	9.4
Mayr *et al.* [49]	2006	Austria	1 yr	Medical-surgical	1	3,347	59.2	28.6	SAPS II	37.6	3	4.3
Priestap and Martin [48]	2006	Canada	Hospital discharge	Medical-surgical	31	47,163	61.7	40.8	APACHE II	15.1	5.3	9.3
Tobin and Santamaria [47]	2006	Australia	Hospital discharge	Medical-surgical	1	10,963	64	35.0	APACHE II	13^b^	N/A	4.4
Fernandez *et al.* [50]	2006	Spain	Hospital discharge	Medical-surgical	1	1,159	60.2	N/A	APACHE II	20^b^	N/A	9.6
				Medical-surgical								
Pilcher *et al.* [46]	2007	Australia/New Zealand	Hospital discharge	Medical-surgical	41	76,690	59	N/A	APACHE III	46.3	5.3	5.8
Song *et al.* [45]	2007	Korea	54.4 mo	N/A	1	1,087	65	N/A	APACHE III	N/A	8.6	N/A
Ho *et al.* [42]	2008	Australia	Hospital discharge	Medical-surgical	1	603	53	N/A	APACHE II	15.7	2	4.3
Gajic *et al.* [44]	2008	USA, Netherlands	7 days	Medical	1	1,242	N/A	45.8	APACHE III	59.2	8.1	0.4
Campbell *et al.* [12]	2008	UK	Hospital discharge	Medical-surgical	1	4,376	63^b^	41.1	APACHE II	19^b^	8.8	11.2
Hanane *et al.* [43]	2008	USA	Hospital discharge	Medical-surgical	3	11,659	62.7	46.8	APACHE III	51.3	9.1	4.5
Kaben *et al.* [41]	2008	Germany	Hospital discharge	Surgical	1	2,852	62	35.9	SAPS I	33.5	13.3	4.8
Laupland *et al.* [40]	2008	Canada	Hospital discharge	Medical-surgical	4	17,864	63.7^b^	26.6	APACHE II	25.1	N/A	6.7
Sakr *et al.* [39]	2008	Europe	60 days	N/A	198	1,729	59.8	39.3	SAPS II	31.4	N/A	7.2
Chrusch *et al.* [38]	2009	Canada	7 days	Medical, Surgical	2	8,222	59.3	N/A	APACHE II	18.6	5.2	0.3
Litmathe *et al.* [37]	2009	Germany	Hospital discharge	Cardiovascular	1	3,374	74.3	30.3	N/A	N/A	5.9	2.1
Fernandez *et al.* [35]	2010	Spain	Hospital discharge	Medical-surgical	31	3587	61.5	33.6	N/A	N/A	4.6	5.9
Al-Subaie *et al.* [36]	2010	UK	14 days	Medical-surgical	1	1,185	60	45.1	APACHE II	16^b^	7	2.9
Utzolino *et al.* [33]	2010	Germany	Hospital discharge	Surgical	1	2,114	62.1	36.4	N/A	N/A	11.8	3.7
Silvestre *et al.* [34]	2010	Portugal	Hospital discharge	Medical-surgical	1	156	55	40.4	APACHE II	14.6	N/A	18.6
				Medical-surgical								
Renton *et al.* [29]	2011	Australia	Hospital discharge	Medical-surgical	97	247,103	59.9	N/A	APACHE III	47	5.5	5.3
Fernandez *et al.* [32]	2011	Spain	Hospital discharge	Medical-surgical	31	201	60.5	31	N/A	N/A	6	22
Kramer *et al.* [11]	2011	USA	Hospital discharge	Medical-surgical	38	229,961	N/A	44.0	N/A	N/A	6	N/A
Silva *et al.* [28]	2011	Brazil	Hospital discharge	Medical-surgical	4	600	60.7	43.3	SAPS II	25.5	9.1	N/A
Laupland *et al.* [31]	2011	France	Hospital discharge	Mixed	N/A	5992	62^b^	39	SAPS II	40^b^	N/A	5.9
Ouanes *et al.* [30]	2012	France	Hospital discharge	Medical-surgical	4	3,462	60.6	38.3	SAPS II	35.1	3.3	3.2
Badawi and Breslow [26]	2012	USA	48 hr/Hospital discharge	Mixed	402	704,963	62.1	45.9	APACHE IV	47	2.5	3.1
Reini *et al.* [21]	2012	Sweden	30 days	Medical-surgical	1	354	60.6	25.4	SAPS III	61^b^	3.7	8.2
Araújo *et al.* [27]	2012	Portugal	Hospital discharge	Medical-surgical	1	296	64.7	43.0	SAPS II	43.7	4.7	22.6
Brown *et al.* [25]	2012	USA	21 days	Medical-surgical	156	196,250	N/A	N/A	MPMO-III	10.9	5.4	N/A
Joskowiak *et al.* [24]	2012	Germany	Hospital discharge	Cardiovascular	1	7,105	69.1	30.7	euroSCORE	9	7.8	1.2
Timmers *et al.* [20]	2012	Netherlands	11 yr	Medical-surgical	1	1,682	58.6	33.3	APACHE II	11.1	8	N/A
Mahesh *et al.* [23]	2012	UK	Hospital discharge	Cardiovascular	1	6,101	N/A	27.8	euroSCORE	7.6	N/A	0.39
Ranzani *et al.* [22]	2012	Brazil	Hospital discharge	Medical	1	409	48.6	49	APACHE II	16	17.4	18.3
Kramer *et al.* [4]	2013	USA	Hospital discharge	Mixed	105	263,082	61.5	N/A	APACHE IV	41.3	6.3	N/A
Yip and Ho [19]	2013	Australia	34 mo	Medical-surgical	1	1,446	50.2	35.7	APACHE II	19^b^	7.3	12.3

^a^APACHE, Acute Physiology and Chronic Health Evaluation; APS, Acute Physiology Score; ICU, Intensive care unit; MICU, Medical intensive care unit; MPMO-III, Mortality Probability Admission Model; N/A, Not available; NICU, Neurosurgical intensive care unit; SAPS, Simplified Acute Physiology Score; SICU, Surgical intensive care unit;. ^b^Median score. ^c^Mixed, MICU, SICU, NICU. ^d^Two Mixed, two SICUs and three MICUs.

**Figure 2:**
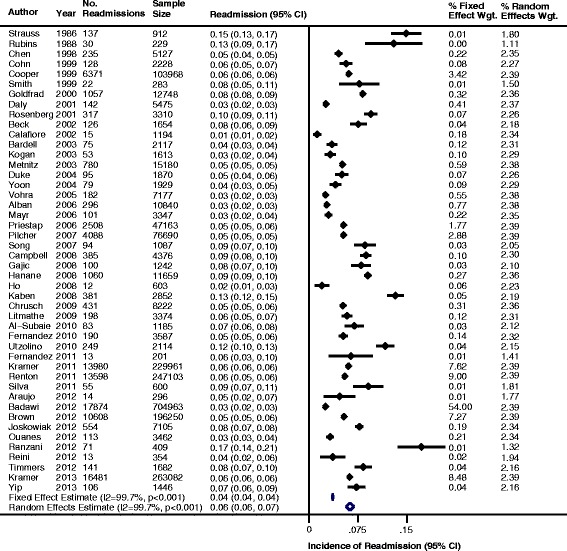
**Incidence of readmission to the intensive care unit (ICU) for patients discharged alive from the ICU.** CI, Confidence interval.

**Figure 3:**
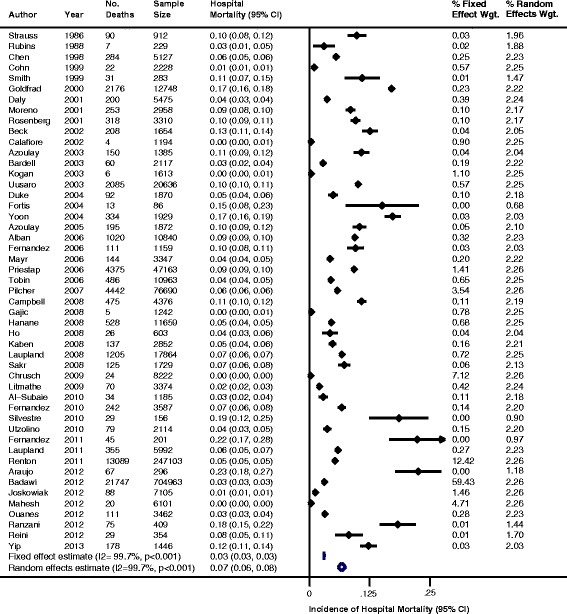
**Incidence of hospital mortality for patients discharged alive from the intensive care unit.** CI, Confidence interval.

The pooled cumulative incidence of readmission to the ICU and cumulative incidence of hospital mortality using both fixed effects models and random effects models are summarized in Figure 2 and Figure 3, respectively. In patients discharged alive from the ICU, the fixed effects pooled cumulative incidence of readmission to the ICU during the same hospitalization was 4.0 readmissions per 100 patient discharges (95% CI, 3.9 to 4.0), whereas the random effects pooled cumulative incidence was 6.3 readmissions per 100 patients (95% CI, 5.6 to 6.9). In patients discharged alive from the ICU, the fixed effects pooled hospital mortality cumulative incidence during the same hospitalization was 3.3 deaths per 100 patient discharges (95% CI, 3.3 to 3.3), whereas the random effects pooled cumulative incidence was 6.8 deaths per 100 patient discharges (95% CI, 6.1 to 7.6). Heterogeneity among these estimates was high, with *I*^2^-values of 99.7% and *P* < 0.001 for all estimates.

The stratified pooled cumulative incidence of readmission to the ICU and the stratified pooled cumulative incidence of hospital mortality for patients discharged alive from the ICU varied by geographic region, ICU type, patient characteristics and study characteristics (Table 2). Compared to medical-surgical ICUs, lower cumulative incidences of readmission (3.8 vs. 5.6 readmissions per 100 patient discharges) and hospital mortality (0.1 vs. 4.4 deaths per 100 patient discharges) were reported for cardiovascular ICUs. The cumulative incidence of ICU readmission and hospital mortality varied according to age, severity of illness and goals of care designations of the patients included in the studies. For example, studies that excluded patients with DNRs had lower cumulative incidences of readmission (3.5 vs. 5.5 readmissions per 100 patient discharges) and hospital mortality (2.2 vs. 3.5 deaths per 100 patient discharges) compared to studies that included DNR patients.

**Table 2:** **Pooled cumulative incidence of ICU readmission and hospital mortality after patient discharge from ICU** ^**a**^

**Variables**	**ICU readmission**				**Hospital mortality**			
	**Studies,** ***n***	**Patients,** ***n***	**Fixed effects pooled proportion (95% CI)**	**Random effects pooled proportion (95% CI)**	**Studies,** ***n***	**Patients,** ***n***	**Fixed effects pooled proportion (95% CI)**	**Random effects pooled proportion (95% CI)**
**Total pooled estimates**	**46**	**2,002,269**	**0.040 (0.039 - 0.040)**	**0.063 (0.056 - 0.069)**	**49**	**1,254,183**	**0.033 (0.033 - 0.033)**	**0.068 (0.061 - 0.076)**
**Geographic region**								
North America	16	1,591,273	0.037 (0.037 - 0.038)	0.064 (0.053 - 0.076)	13	815,876	0.030 (0.029 - 0.030)	0.050 (0.036 - 0.065)
Europe	20	77,646	0.048 (0.047 - 0.049)	0.062 (0.050 - 0.074)	27	95,681	0.025 (0.024 - 0.026)	0.081 (0.064 - 0.098)
Australia / New Zealand	5	327,712	0.054 (0.054 - 0.055)	0.051 (0.047 - 0.056)	6	338,675	0.054 (0.053 - 0.055)	0.057 (0.051 - 0.063)
Other regions	5	5,638	0.049 (0.043 - 0.054)	0.081 (0.050 - 0.111)	3	3,951	0.010 (0.007 - 0.013)	0.119 (0.000 - 0.256)
**ICU type**								
Medical-surgical ICU	28	883,365	0.056 (0.055-0.056)	0.058 (0.054 - 0.061)	29	471,305	0.044 (0.044 - 0.045)	0.086 (0.073 - 0.099)
Cardiovascular ICU	6	23,195	0.038 (0.035-0.040)	0.044 (0.024 - 0.065)	6	22,119	0.007 (0.006 - 0.008)	0.012 (0.006 - 0.019)
Other ICU types	12	1,095,709	0.032 (0.032-0.033)	0.081 (0.065 - 0.096)	14	760,759	0.031 (0.031 - 0.032)	0.066 (0.049 - 0.082)
**Patient characteristics**								
Age <60	16	376,251	0.054 (0.053 - 0.054)	0.065 (0.057 - 0.072)	18	378,326	0.041 (0.041 - 0.042)	0.092 (0.075 - 0.109)
Age >60	29	1,624,824	0.038 (0.037 - 0.038)	0.062 (0.053 - 0.070)	28	865,604	0.033 (0.032 - 0.033)	0.060 (0.049 - 0.070)
SOI predicted <10% mortality	3	3,369	0.086 (0.077 - 0.095)	0.086 (0.077 - 0.095)	2	9,059	0.005 (0.003 - 0.006)	0.044 (0.000 - 0.125)
SOI predicted >10% mortality	31	1,534,181	0.036 (0.036 - 0.037)	0.064 (0.056 - 0.072)	39	1,228,973	0.035 (0.035 - 0.036)	0.076 (0.067 - 0.084)
**Study characteristics**								
DNR patients excluded	13	1,372,056	0.035 (0.035 - 0.035)	0.068 (0.056 - 0.080)	14	1,132,425	0.022 (0.021 - 0.023)	0.057 (0.045 - 0.070)
DNR patients included	33	630,213	0.055 (0.055 - 0.056)	0.059 (0.054 - 0.064)	35	121,758	0.035 (0.035 - 0.035)	0.076 (0.064 - 0.089)
High study quality	36	1,643,624	0.037 (0.037 -0.037)	0.066 (0.058 - 0.073)	40	1,215,780	0.033 (0.033 - 0.033)	0.071 (0.063 - 0.079)
Low study quality	10	358,645	0.058 (0.057-0.059)	0.052 (0.043 - 0.061)	9	38,403	0.034 (0.032 - 0.036)	0.062 (0.033 - 0.091)
Adjusted for confounding factors	41	1,618,703	0.037 (0.036 - 0.037)	0.060 (0.054 - 0.067)	43	1,231,324	0.034 (0.034 - 0.035)	0.065 (0.057 - 0.072)
Not adjusted for confounding factors	5	383,566	0.063 (0.062 - 0.064)	0.076 (0.069 - 0.082)	6	22,859	0.013 (0.011 - 0.014)	0.110 (0.034 – 0.186)
Follow-up >21 days	41	1,090,407	0.057 (0.057 - 0.058)	0.061 (0.057 - 0.065)	45	538,571	0.045 (0.044 - 0.045)	0.076 (0.066 - 0.086)
Follow-up <21 days	5	911,862	0.029 (0.029 - 0.029)	0.056 (0.037 - 0.074)	4	715,612	0.028 (0.027 - 0.028)	0.016 (0.000 - 0.036)
Patient number >1000	37	1,998,382	0.040 (0.039 - 0.040)	0.060 (0.053 - 0.067)	39	1,250,654	0.033 (0.033 - 0.033)	0.060 (0.052 - 0.068)
Patient number <1000	9	3,887	0.058 (0.051 - 0.065)	0.086 (0.046 - 0.126)	10	3,529	0.085 (0.076 - 0.094)	0.129 (0.089 - 0.168)
Multiple ICU study	19	1,934,123	0.040 (0.039 - 0.040)	0.051 (0.035 - 0.066)	20	1,177,518	0.035 (0.035 – 0.036)	0.076 (0.064 - 0.087)
Single ICU study	27	68,146	0.041 (0.040 - 0.043)	0.063 (0.059 - 0.067)	29	76,665	0.017 (0.016 - 0.018)	0.064 (0.053 - 0.075)

^a^CI, Confidence interval; DNR, Do-not-resuscitate order; ICU, Intensive care unit; SOI, Severity of illness.

## Discussion

In this meta-analysis, we report the first pooled estimates of readmission to the ICU and hospital mortality for patients discharged alive from the ICU. These estimates suggest that, on average, for every 100 patients discharged alive from the ICU, between 4 and 6 patients will be readmitted to the ICU and between 3 and 7 patients will die prior to hospital discharge. Important variations in the incidence of readmission and mortality were observed according to geographic regions and patient-related, institutional and study methodological characteristics.

Our study underscores important opportunities and challenges in improving the quality of care provided to patients discharged from intensive care. We identified estimates of readmission and death for patients discharged alive from the ICU that are similar in magnitude to the estimates of adverse events reported in an Institute of Medicine report, *To Err Is Human*, that prompted major efforts to improve the safety and quality of care [71,72]. Although readmission to the ICU and hospital mortality after ICU discharge do not equate to medical errors or adverse events and are not necessarily preventable [12], our data highlight that patient discharge from the ICU is a high-risk transition of care. There are opportunities to reduce the risks pertaining to patients (for example, relapsing and/or remitting comorbid illness), providers (for example, differential continuity of care), institutions (for example, availability of transition resources) and health systems (for example, ICU capacity) [73]. Our analysis reinforces the importance of measuring performance and considering internal (that is, monitoring performance over time) and external (that is, monitoring performance across institutions) benchmarking to guide quality improvement activities. For example, deviations from anticipated performance could be used to trigger audits of patient care to identify potentially preventable events and their root causes and thereby implement locally tailored interventions.

Our results can be used to inform quality metrics designed to measure the incidence of readmission to the ICU and the incidence of hospital mortality after patient discharge from the ICU. Currently, there is no consensus on ICU benchmarks for readmission and post-ICU mortality. ICU readmission was initially identified by Cooper *et al*. as an important indicator that captured complementary aspects of hospital-related performance [8]. Rosenberg *et al*. identified a readmission incidence of 7% and suggested its use as a quality-of-care indicator [5]. More recently, professional societies [6], provider groups [74] and accreditation organizations [75] across multiple countries [76] have proposed ICU readmission as a quality indicator, but they have not specified benchmark values. Measures of ICU and hospital mortality have similarly been proposed [10,76]. Systematic reviews and meta-analyses have been used to derive quality improvement benchmarks [77], and our present study provides literature-based estimates of readmission to the ICU and hospital mortality that could be used by institutions to select potential benchmark values.

So, which literature-based estimates should be considered? Our analyses provide two sets of pooled estimates for both ICU readmission and hospital mortality that offer a range of potential benchmarks. The fixed effects model assumes that ICU readmission incidence is the same from study to study and provides a weighted average that gives large studies greater weight [78]. The random effects model does not assume that the ICU readmission incidence is the same from study to study (that is, that it may vary from study to study) and provides a weighted average that gives studies of different sizes similar weights [79]. Although the random effects model does better justice to the full range of data available, it does potentially allow a larger weight to be given to smaller studies that may have been selected for publication on the basis of their higher event rates [18]. Therefore, one approach would be to consider ICU readmission incidence (6 patients per 100 patient discharges) and hospital mortality incidence (7 patients per 100 patient discharges) above the random effects estimates to represent suboptimal quality of care. To represent adequate quality of care accurately, it may be necessary to consider ICU readmission incidence (4 to 6 patients per 100 patient discharges) and hospital mortality incidence (3 to 7 patients per 100 patient discharges) using both the fixed effects and random effects models. It may also be necessary to consider ICU readmission incidence (4 patients per 100 patient discharges) and hospital mortality incidence (3 patients per 100 patient discharges) below the fixed effects estimates as high-quality care and benchmark targets. The stratified analyses can be used to further refine benchmark selection to more closely represent different organizations’ patient and institutional characteristics. As an important caveat, the data highlight the complexity of identifying appropriate benchmarks, reinforce the importance of a cautious approach to adopting benchmarks and suggest potential value in employing benchmark ranges as opposed to individual values in quality improvement initiatives.

Our data also highlight that hospital mortality is common among patients discharged from the ICU. This reinforces observations that the utilization of intensive care resources by patients with life-limiting illnesses is steadily rising and that end-of-life care is increasingly initiated in the ICU [80,81]. Whereas many of these patients will die during their ICU stay, others will be discharged from the ICU before dying. This suggests that consideration needs to be given to ensure that end-of-life care is effectively delivered during transitions of care. Incorporating joint metrics for goals of care reconciliation at the time of patient discharge from the ICU, as well as both ICU readmission and hospital mortality following patient discharge from the ICU, may help in the evaluation and monitoring of the care provided to patients discharged from the ICU who are at the end of life [82].

There are caveats to our study findings. First, the studies included in this analysis were identified by conducting a literature search targeted for studies in which associations between prognostic factors and the risk of readmission to ICU and hospital mortality for patients discharged alive from the ICU were examined. Nevertheless, it is unlikely that the incidence in other studies reporting readmission and death after patient discharge would be different from ours. Second, we identified heterogeneity that is not fully explained. This is an expected finding, given the diversity of geographic locations (for example, health systems, available resources), institutions (for example, procedures for discharge and post-ICU care), providers (for example, discharge practices) and patient populations (for example, severity of illness, patient and family care preferences) in the included studies. We have discussed the relative merits and limitations of using fixed effects models and random effects models to interpret benchmarks. Against this backdrop of heterogeneity, our meta-analysis summarizes what other institutions are reporting. Third, in the majority of studies, patients were followed to hospital discharge and data at fixed time periods following patient discharge from the ICU were not reported. Although measuring readmission to the ICU and hospital mortality during the remainder of a patient’s hospital stay provides valuable information, the implications of these events likely vary by time period (that is, implication of patient readmission within 24 hours is likely different from readmission within 7 days [15]) and may introduce bias into external benchmarking activities if the hospitals being compared employ different discharge practices (for example, timing of discharge or disposition to home, to rehabilitation, to long-term care [83]). Establishing consensus time periods for measuring quality metrics of transitions of patient care between the ICU and hospital ward would facilitate future research and quality improvement initiatives.

## Conclusions

On the basis of our analysis of the literature, for every 100 patients discharged alive from the ICU, on average, between 4 and 6 patients will be readmitted to the ICU and between 3 and 7 patients will die prior to hospital discharge. Opportunities exist to improve the quality of care provided to patients discharged from intensive care. The literature-based estimates derived from this systematic review and meta-analysis can be used to inform the selection of benchmarks for quality metrics of transitions of patient care between the ICU and the hospital ward.

## Key messages

- The discharge of patients from the ICU to a hospital ward is a vulnerable period in health care delivery.
- Estimates suggest that for every 100 patients discharged alive from the ICU, on average, between 4 and 6 patients will be readmitted to the ICU and between 3 and 7 patients will die prior to hospital discharge.
- The literature-based estimates derived from this systematic review and meta-analysis can be used to inform the selection of benchmarks for quality metrics of transitions of patient care between the ICU and the hospital ward.

## Abbreviations

APACHE: Acute Physiology and Chronic Health Evaluation
APS: Acute Physiology Score
CI: Confidence interval
DNR: Do not resuscitate
ICU: Intensive care unit
MICU: Medical intensive care unit
MPMO-III: Mortality Probability Admission Model
NICU: Neurosurgical intensive care unit
SAPS: Simplified Acute Physiology Score
SICU: Surgical intensive care unit
SOI: Severity of illness
